# 25‐Hydroxycholesterol protects against acute lung injury via targeting MD‐2

**DOI:** 10.1111/jcmm.13820

**Published:** 2018-08-09

**Authors:** Wei Ouyang, Hui Zhou, Chao Liu, Shiwei Wang, Yu Han, Jingyan Xia, Feng Xu

**Affiliations:** ^1^ Department of Infectious Diseases The Second Affiliated Hospital Zhejiang University School of Medicine Hangzhou China; ^2^ Experimental Medical Class 1102 Chu Kochen Honor College Zhejiang University Hangzhou China; ^3^ School of Life Sciences Peking University Beijing China; ^4^ Department of Radiation Oncology The Second Affiliated Hospital Zhejiang University School of Medicine Hangzhou China

**Keywords:** 25‐hydroxycholesterol, acute lung injury, inflammatory response, myeloid differentiation protein 2, Toll‐like receptor 4

## Abstract

Acute lung injury (ALI) is mainly caused by uncontrolled inflammatory response, and it remains without effective therapeutic options. 25‐hydroxycholesterol (25HC) has been reported to be a potent regulator of inflammation. The aim of this study was to investigate the effects of 25HC on lipopolysaccharide (LPS)‐induced ALI. C57BL/6 mice were pretreated with 25HC intraperitoneally before intratracheal exposure to LPS. Our results showed that 25HC pretreatment improved survival rate, attenuated the pathological changes of the lung and decreased the release of inflammatory cytokines in mice. Consistently, 25HC reduced expression of Toll‐like receptor (TLR4)‐mediated inflammatory cytokines in vitro. These effects of 25HC were obtained by preventing LPS binding to TLR4 via interaction with myeloid differentiation protein 2 (MD‐2). Crystal structure analysis suggested that 25HC could bind MD‐2 with high affinity into its hydrophobic pocket. Furthermore, LPS‐induced activation of Akt/NF‐κB pathway was partially down‐regulated by 25HC pretreatment. In summary, this study demonstrates that 25HC could inhibit the overwhelming inflammatory response through MD‐2 interaction, which suppresses Akt/NF‐κB signalling pathway. These findings suggest 25HC may be a promising candidate for ALI prevention.

## 1 INTRODUCTION

Acute lung injury (ALI) is recognized as refractory hypoxaemia, noncardiogenic oedema, decreased lung compliance and diffuse pulmonary infiltrates, which often result in respiratory failure.^1^ The pathophysiological mechanisms of ALI are related to overwhelming innate immune response, leading to acute organ failure including the lungs.^2^ There are no specific pharmacological therapies for ALI and other therapeutic approaches are extremely limited so far.^3^, ^4^

Severe pneumonia is considered as a main cause of ALI.^5^ Lipopolysaccharide (LPS) is a component of Gram‐negative bacteria that cause severe diseases including life‐threatening pneumonia. Toll‐like receptors (TLRs) sense microbial infections via interaction with pathogen‐associated molecules in innate immunity system.^6^, ^7^ TLR4 recognizes the lipid chains of LPS through mediation of an accessory protein, myeloid differentiation protein 2 (MD‐2).^8^, ^9^ MD‐2 is a small extracellular glycoprotein which interacts with most of lipid chains of LPS through its hydrophobic pocket.^10^, ^11^ The interaction of LPS and MD‐2 bridges two TLR4 molecules to induce the multimerization of the LPS‐TLR4‐MD‐2 complex.^12^ Activation of the TLR4‐MD‐2 complex leads to the transcription of nuclear factor (NF)‐κB, which triggers the overproduction of proinflammatory cytokines, such as interleukin (IL)‐6, tumour necrosis factor α (TNF‐α) and IL‐8 in the early stage of immune response.^13^, ^14^ The cytokine‐mediated inflammation prompts the progression of ALI.^15^

Oxysterols, oxidized forms of cholesterol, participate in cholesterol homeostasis, immune response and pathogenesis of several diseases such as neurodegenerative diseases and atherosclerosis.^16^ 25‐hydroxycholesterol (25HC), one of the oxysterols, has recently garnered much attention as a potential regulator of innate immunity and inflammation.^17^ Previous studies have demonstrated 25HC possesses antiviral effects against various viruses, including vesicular stomatitis virus (VSV), human immunodeficiency virus (HIV), Ebola virus (EBOV) and Zika virus (ZIKV) through inhibition of viral entry.^18^, ^19^, ^20^ Moreover, 25HC has been reported to mediate the activation of NF‐κB signalling to induce IL‐6 and IL‐8 expression.^21^, ^22^ A recent study observed that 25HC decreased the production of IL‐1β and inflammasome activity, leading to improve recovery from septic shock.^23^ These findings suggested that 25HC could be involved in the regulation of inflammatory processes and its function varies based on biological conditions.

In this study, we aimed to investigate the effects of 25HC on LPS‐treated ALI murine model and RAW264.7 cells. Our study reveals 25HC to possess anti‐inflammatory role in both mouse and cell models, which collectively indicate 25HC as a potential therapeutic option for ALI and other acute inflammatory diseases.

## 2 MATERIALS AND METHODS

### 2.1 Reagents

25HC, LPS (*E. coli* O111:B4) and fluorescein isothiocyanate‐labelled LPS (FITC‐LPS) were purchased from Sigma‐Aldrich (St. Louis, MO, USA). Recombinant human MD‐2 (rhMD‐2) protein was obtained from R&D Systems (Minneapolis, MN, USA). Biotin‐LPS was purchased from Invivogen (San Diego, CA, USA). Anti‐MD‐2 antibody was from Abcam (Cambridge, UK). Antibodies against phospho‐Akt, phospho‐NF‐κB p65, phospho‐JNK mitogen‐activated protein kinase (MAPK), phospho‐Erk42/44 MAPK, phospho‐p38 MAPK, Akt, NF‐κB p65, MAPKs of JNK, Erk42/44, p38, and β‐actin were purchased from Cell Signaling Technology (Boston, MA, USA).

### 2.2 Mouse ALI model

The animal studies were approved by the Ethics Committee of animal experiments at Zhejiang University, and all the processes are in strict accordance with the National Institutes of Health Guide for the Care and Use of Laboratory Animals. Pathogen free, female C57BL/6 mice (6‐8 weeks old) were obtained from the Animal Center of Slaccas (Shanghai, China). The mice were injected intraperitoneally with 25HC (50 mg/kg) or the vehicle control 6 hours before intratracheal stimulation of LPS (3 mg/kg). The mice treated with vehicle alone were employed as control. Six hours after LPS treatment, the mice were killed to evaluate histological changes and inflammatory cytokine expression. For the mortality study, mice were intratracheally challenged with a lethal dose of LPS (40 mg/kg). The survival rate was recorded every 12 hours until 3 days post‐LPS injection.

### 2.3 Histological analysis

Whole lung tissues of mice were fixed with 4% paraformaldehyde neutral buffer overnight, embedded in paraffin, sectioned at 4 μm thickness and stained with Haematoxylin‐Eosin (H&E). Morphometric assessment was conducted under an automatic photo‐microscope (Olympus, Tokyo, Japan).

### 2.4 Collection and analysis of bronchoalveolar lavage fluid (BALF)

BALF was collected through lavaging the lungs using a tracheal cannula with 1 mL of ice‐cold PBS for three times. After removing the erythrocytes in the BALF by lysis buffer, the total cell number was counted. 2 × 10^5^ cells were smeared on a slide and stained with Giemsa regent (Nanjing Jiancheng Bio‐engineering Institute, Nanjing, China) for cell differentiation. BALF protein concentrations were determined using the bicinchoninic acid (BCA) protein assay kit (Beyotime Biotechnology, Beijing, China). LDH activity in BALF was determined with LDH Cytotoxicity Assay Kit (Nanjing Jiancheng Bio‐engineering Institute).

### 2.5 Enzyme‐linked immunosorbent assay (ELISA)

The levels of myeloperoxidase (MPO) (Cloud‐Clone Corp, Wuhan, China), IL‐1β, IL‐6, TNF‐α (Invitrogen, Carlsbad, CA, USA), keratinocyte‐derived cytokine (KC), and macrophage inflammatory protein‐2 (MIP‐2) (MultiSciences, Hangzhou, China) in the cell‐free BALF were measured using ELISA kits according to the manufacturer's instructions.

### 2.6 Cell culture

RAW264.7 mouse macrophages (American Type Culture Collection) were cultured in Dulbecco's modification of Eagle's medium (DMEM) containing 2 mmol/L glutamine, 10% foetal bovine serum, 100 U/mL of penicillin and 100 μg/mL of streptomycin at 37°C in an atmosphere with 5% CO_2_.

### 2.7 Real‐time quantitative PCR (RT‐qPCR)

Total RNAs were extracted with the Ultrapure RNA kit (Cwbiotech, Beijing, China) and reverse‐transcribed to cDNA using cDNA synthesis system (Applied Biosystems, Foster City, CA, USA). The qPCR was performed using SYBR Green PCR Master Mix (Cwbiotech) in the CFX96 Touch Real‐Time PCR Detection System (Bio‐Rad, Hercules, CA, USA). The primer sequences are listed as Table 1. β‐actin was amplified as a housekeeping gene. The relative expression levels of target genes were calculated by applying ΔΔCt (cycle threshold) approach.

**Table 1:** Primers sequences for qPCR detection

Gene	Forward primers (5′‐3′)	Reverse primers (5′‐3′)
β‐actin	GTATCCTGACCCTGAAGTACC	GAAGGTCTCAAACATGATCT
IL‐1β	CCTCCTTGCCTCTGATGG	AGTGCTGCCTAATGTCCC
IL‐6	AGTTGCCTTCTTGGGACTGA	TCCACGATTTCCCAGAGAAC
TNF‐α	CTGGGACAGTGACCTGGACT	GCACCTCAGGGAAGAGTCTG
KC	ACCCAAACCGAAGTCATA	AGGTGCCATCAGAGCAGT
MIP‐2	CCCAGACAGAAGTCATAGC	TCCTTTCCAGGTCAGTTA

### 2.8 ELISA for LPS binding to MD‐2

ELISA was used for assaying LPS binding to rhMD‐2 as described before.^24^ Briefly, the anti‐MD2 antibody was coated in 96‐well micro‐plates for overnight at 4°C. The plate was washed and blocked with 2% bovine serum albumin (BSA) for 2 hours at room temperature. RhMD‐2 (0.1 μmol/L) in 10 mmol/L Tris‐HCl buffer (pH 7.4) was added to the plate for 2 hours. Then, biotin‐LPS (100 ng/mL) was incubated with or without 25HC (1 μmol/L to 20 μmol/L) for 30 minutes before streptavidin conjugated to horseradish peroxidase (MultSciences) was added for 1 hour. After washing, tetramethylbenzidine (TMB) used to determine the activity of horseradish peroxidase. The absorbance values of each well were measured at 450 nm.

### 2.9 Fluorescence spectroscopy detection

All the measurements of fluorescence spectra were performed on FluoroMax^®^‐4 spectrofluorometer (Horiba Jobin Yvon IBH Ltd., Glasgow, UK) equipped with a 1‐cm quartz cell at 25°C. The slit widths of both excitation and emission were set at 4 nm. RhMD‐2 at 5 nmol/L and 25HC (1, 5, 10, or 20 μmol/L) were mixed in PBS (pH 7.4) and incubated 30 minutes to reach stable relative fluorescence units. The emission spectra of intrinsic fluorescence of MD‐2 were recorded from 420 nm to 540 nm under an excitation wavelength of 380 nm.

### 2.10 Flow cytometry analysis

RAW264.7 cells were incubated with vehicle, 0.1% ethanol (EtOH), or different doses of 25HC for 2 hours, washed with PBS twice, and then mixed with 2 μg/mL FITC‐LPS for 20 minutes. The LPS binding of the cells was analysed via a flow cytometry (Beckman Coulter, Inc., CA, USA).

### 2.11 Confocal immunofluorescence microscopy

RAW264.7 cells were grown on glass cover slips. For detection of LPS/MD‐2 colocalization, cells were pretreated with 10 μmol/L 25HC for 2 hours, and then incubated with Alexa Fluor 568‐conjugated LPS (6 μg/mL) (Invitrogen) for 15 minutes. The cells were fixed with 4% paraformaldehyde and blocked with 5% BSA for 1 hour. They were then incubated with anti‐MD‐2 antibody in blocking buffer overnight at 4°C, reacted with Alexa Fluor 488‐conjugated anti‐rabbit IgG secondary antibody (Invitrogen) and mounted with antifluorescence quenching medium. For nuclear translocation of NF‐κB, the cells were pretreated with or without 25HC (10 μmol/L) for 2 hours before 100 ng/mL LPS stimulation. After 1 hour of LPS treatment, cells were washed three times with PBS, followed by fixation with 4% paraformaldehyde for 15 minutes. Then, they were permeabilized with 0.2% Triton X‐100 and blocked with 5% BSA. The cells were incubated with NF‐κB p65 antibody overnight at 4 °C and incubated with Alexa Fluor 594‐labelled secondary antibody (Proteintech, Wuhan, China) at room temperature for 1 hour. Afterwards, nuclei were stained with 1 mg/mL 4′, 6‐diamidino‐2‐phenylindole (DAPI) solution (Solarbio, Beijing, China) for 5 minutes. All slides were visualized and imaged under FV3000 fluorescent microscope (Olympus).

### 2.12 Molecular docking analysis

Crystal structure of human MD‐2 in complex with lipid IVa was retrieved from Protein Data Bank (PDB: 2E59) and docking conformation of 25HC was obtained from PubChem compound database (PubChem CID: 65094). Docking simulation of 25HC with MD‐2 was conducted by AutoDock4.2.6 program (Scripps Research Institute, San Diego, CA USA). The interaction between MD‐2 complex and 25HC was prepared following default protocols of AutoDock Tools. The ligand‐binding groove on the MD‐2 complex was kept rigid, whereas all rotatable bonds of 25HC were set free to allow flexible docking. The centre of macromolecule was picked as the centre of grids which were defined as a box of 30 Å × 30 Å × 30 Å with 0.375 Å spacing. Two hundred docking calculations were performed via Lamarckian Genetic Algorithm local search method. Finally, the docked conformation with lowest docking energy was selected as the final result.

### 2.13 Western blot

Whole cell lysate protein was collected from RAW264.7 cells with 1× RIPA lysis buffer containing 1 mmol/L phenylmethylsulfonyl fluoride and phosphatase inhibitor cocktail tablets (Roche, Basel, Switzerland). Protein concentrations were determined by the BCA Protein Assay reagent (Beyotime Biotechnology). Thirty μg protein of cell lysates was loaded on 10% SDS‐PAGE gel, separated by electrophoresis and transferred onto PVDF membrane (Millipore, Billerica, MA, USA). After blocking in Tris‐buffered saline Tween‐20 with 5% fresh nonfat milk, the membranes were probed with indicated primary antibody overnight at 4°C. The membranes were incubated with horseradish peroxidase‐conjugated goat anti‐rabbit IgGs, and the probed bands were visualized by chemiluminescence system (Syngene, Cambridge, UK).

### 2.14 Statistical analysis

Quantitative data are shown as mean ± SEM and calculated on GraphPad Prism 5. Statistical significance among multiple groups was performed using one‐way ANOVA test. Mouse survival curve analysis was evaluated by the log rank test. A *P* value less than 0.05 was considered to be statistically significant.

## 3 RESULTS

### 3.1 25HC pretreatment reduces mortality and lung injury in LPS‐induced ALI mice

**Figure 1:**
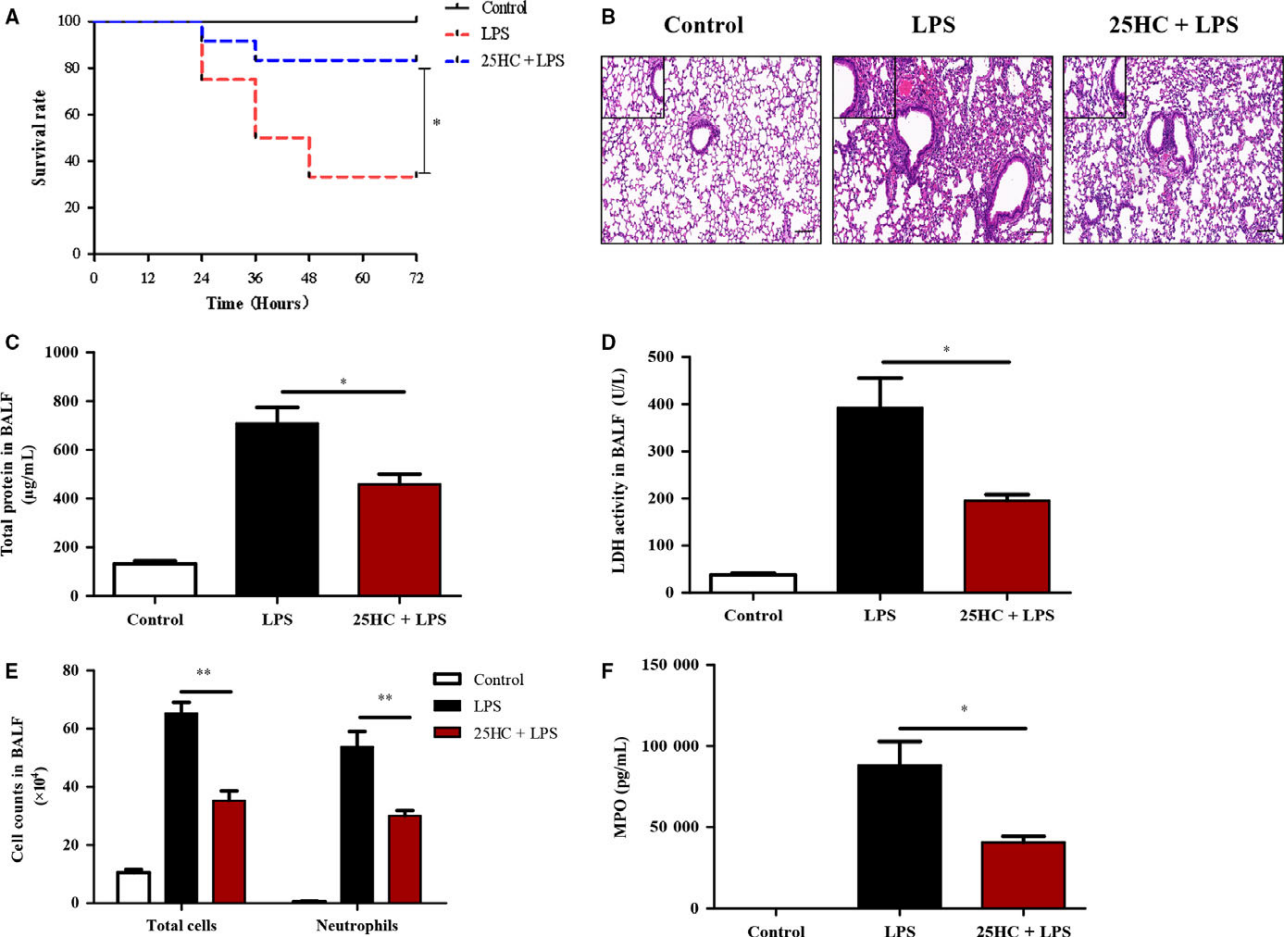
25HC pretreatment reduces mortality and lung injury in LPS‐induced ALI mice. (A) C57BL/6 mice were received with 25HC (50 mg/kg) or vehicle by intraperitoneal injection 6 h before intratracheal stimulation of LPS (40 mg/kg). Mouse survival rate was observed every 12 hours up to 72 hours (n = 12 mice/group). (B‐F) C57BL/6 mice were intraperitoneally received with 25HC (50 mg/kg) or vehicle 6 h before intratracheal administration of LPS (3 mg/kg). Then, the mice were killed 6 h after LPS treatment. (n = 4 mice/group). Representative histopathologic sections of lung tissues by haematoxylin and eosin staining (small panels, ×400, scale bar: 20 μm; big panels, ×100, scale bar: 100 μm) (B). Total protein levels (C), LDH activity (D), counts of the total cells and neutrophils (E) and MPO quantity (F) in BALF were analysed. Quantitative data are shown as mean ± SEM . *P < 0.05 and **P < 0.01

To determine the effect of 25HC on the mortality in ALI, mice were treated with a lethal dose of LPS (40 mg/kg) after administration with 25HC or vehicle. Pretreatment with 25HC markedly improved the survival rate in the LPS‐induced ALI mice (Figure 1A). Furthermore, pulmonary histopathological examinations 6 hours after LPS challenge showed that a low dose of LPS (3 mg/kg) instillation induced significant morphological damages, including alveolar oedema, congestion and infiltration of inflammatory cells in the lung tissue of mice. However, these alterations were remarkably reduced by 25HC pretreatment (Figure 1B). In addition, the concentrations of both total proteins and LDH, the indicators of alveolar capillary barrier and cellular damage, were significantly decreased in the BALF of 25HC‐pretreated ALI mice, compared with those in the ALI group receiving vehicle pretreatment (Figure 1C,D). Similarly, the number of total cells and neutrophils and levels of MPO, a marker of activated neutrophils,^25^ in the BALF of 25HC‐pretreated ALI mice were markedly decreased compared to those in LPS‐treated mice (Figure 1E,F). Taken together, these data demonstrate that 25HC possesses a preventive activity against the LPS‐induced ALI.

### 3.2 25HC inhibits production of proinflammatory cytokines and chemokines in ALI mice

**Figure 2:**
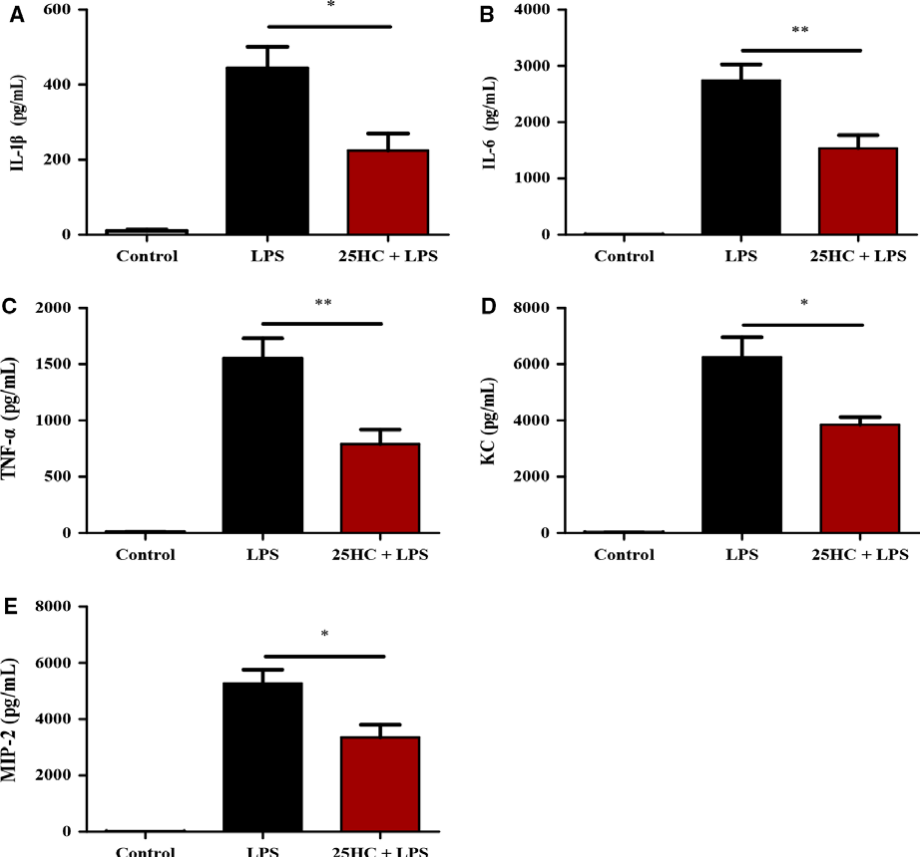
25HC inhibits the production of proinflammatory cytokines and chemokines in ALI mice. C57BL/6 mice were pretreated with either vehicle or 25HC (50 mg/kg) intraperitoneally 6 h before intratracheal challenge with LPS (3 mg/kg) (n = 4 mice per group). The BALF from lungs was collected 6 h after LPS instillation. The concentrations of proinflammatory cytokines including IL‐1β (A), IL‐6 (B), TNF‐α (C) and chemokines such as KC (D) and MIP‐2 (E) in BALF were detected by ELISA. Data are shown as mean ± SEM .*P < 0.05 and **P < 0.01

Proinflammatory cytokines and chemokines contribute to inflammatory responses in ALI.^2^ In this study, the levels of proinflammatory cytokines and chemokines, including IL‐1β, IL‐6, TNF‐α, KC and MIP‐2, in BALF were significantly elevated in mice 6 hours after LPS treatment, compared with those in control. Pretreatment with 25HC markedly reduced LPS‐induced inflammatory responses (Figure 2A‐E).

### 3.3 25HC suppresses LPS‐induced cytokines expression in RAW264.7 cells

**Figure 3:**
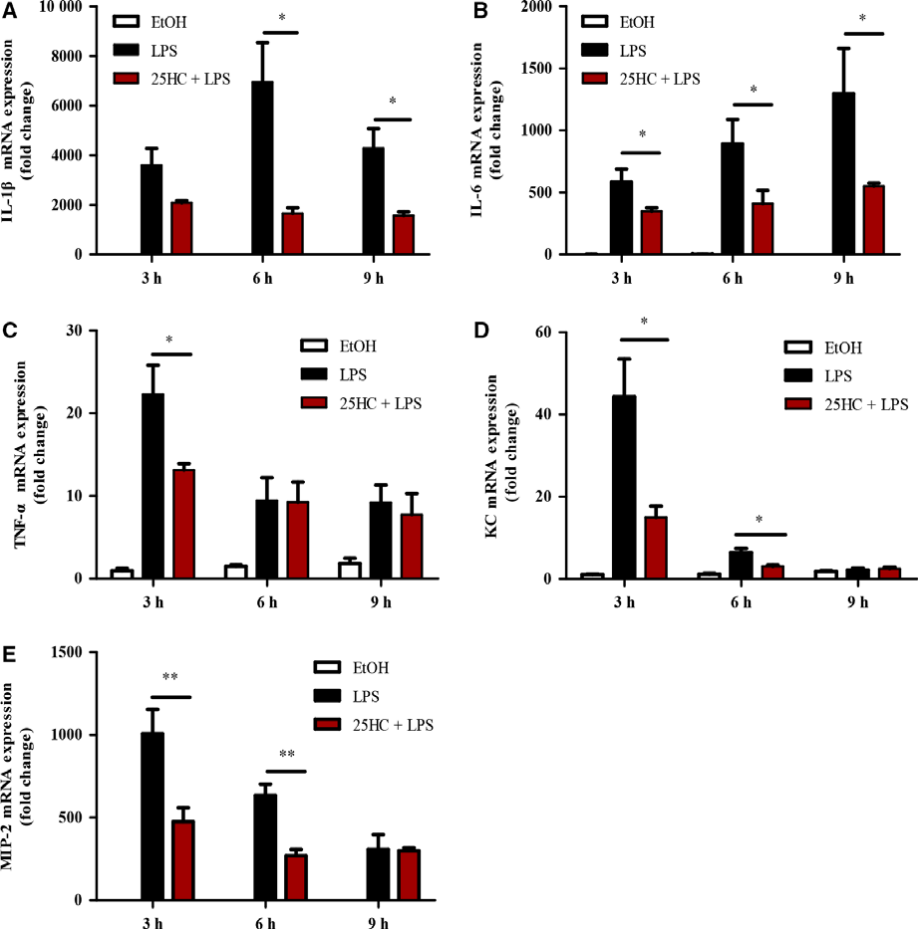
25HC suppresses LPS‐induced cytokines expression in RAW264.7 cells. RAW264.7 cells were pretreated with 25HC (10 μmol/L) or the vehicle, 0.1% ethanol (EtOH), 2 h prior to stimulation with LPS (100 ng/mL) for the indicated times. Relative mRNA expression levels of IL‐1β (A), IL‐6 (B), TNF‐α (C), KC (D) and MIP‐2 (E) was assayed by RT‐qPCR. Data are shown as mean ± SEM .*P < 0.05 and **P < 0.01

To explore the anti‐inflammatory effects of 25HC in vitro, the mRNA levels of IL‐1β, IL‐6, TNF‐α, KC and MIP‐2 were measured by RT‐qPCR. The data showed that 100 ng/mL LPS stimulation significantly induced up‐regulation of the proinflammatory cytokines and chemokines, whereas 10 μmol/L 25HC pretreatment significantly inhibited expression of those cytokines in RAW264.7 cells (Figure 3A‐E).

### 3.4 25HC prevents LPS binding to TLR4 complex via interaction with MD‐2

**Figure 4:**
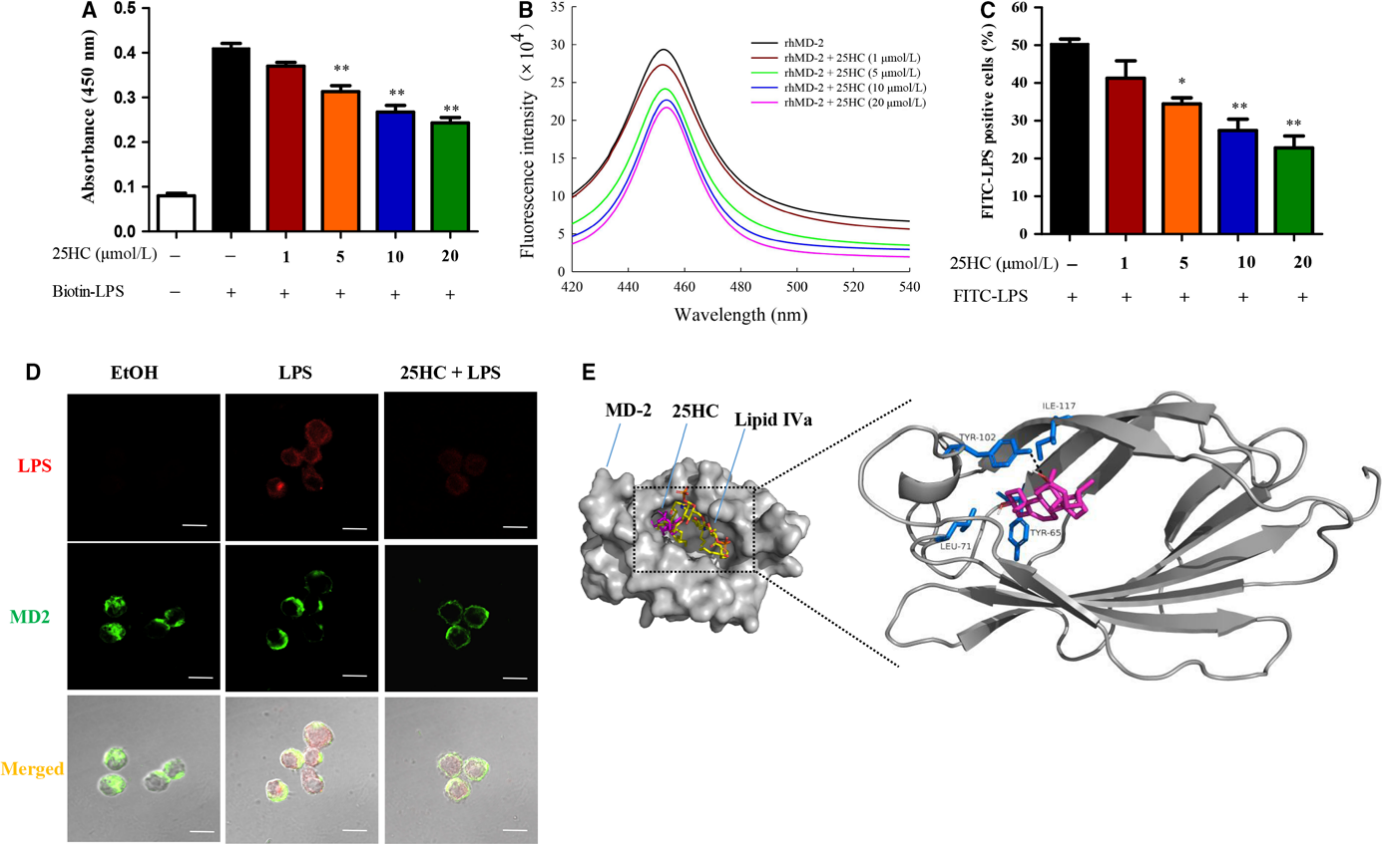
25HC prevents LPS binding to TLR4 receptor through interacting with MD‐2. (A) After anti‐MD‐2 antibody was coated in 96‐well plates for overnight at 4 °C, the rhMD‐2 (0.1 μmol/L) and biotin‐LPS (100 ng/mL) were added into the wells in the presence or absence of 25HC (1, 5, 10, or 20 μmol/L) for 30 min. The binding of LPS to MD‐2 was indicated as absorbance values at 450 nm by ELISA. **P < 0.01 vs biotin‐LPS group. (B) The fluorescence emission spectra of rhMD‐2 (5 nmol/L) were detected with or without 25HC (1, 5, 10, or 20 μmol/L). (C) RAW264.7 cells were pretreated at various concentrations of 25HC (1, 5, 10, or 20 μmol/L) for 2 h and then treated with FITC‐LPS (2 μg/mL) for 20 min. The cells were analysed by flow cytometry. *P < 0.05 and **P < 0.01 vs FITC‐LPS group. (D) RAW264.7 cells were pretreated with either 25HC (10 μmol/L) or 0.1% ethanol (EtOH) for 2 h and then treated with Alexa Fluor 568‐conjugated LPS (6 μg/mL) for 15 min. Confocal immunofluorescence microscopy was performed for analysis. Scale bar, 10 μm. (E) Molecular docking of 25HC with MD‐2 protein was conducted by AutoDock program. 25HC in purple red and lipid IVa in yellow were represented with sticks. MD‐2 was shown as the solid surface (left). The refined model was rendered as 25HC by purple red and its interacting residues on MD‐2 by dark blue. A hydrogen bond between 25HC and Tyr‐102 residue of MD‐2 was depicted as a dotted line (right). Quantitative data are shown as mean ± SEM

During the process of immune response, LPS triggers a series of protein interactions to form a TLR4 complex that includes LPS, LPS‐binding protein (LBP), CD14 and MD‐2.^26^ To further explore the molecular mechanism of the inhibitory action of 25HC on LPS‐induced TLR4 signalling, we investigated whether 25HC interfered with the interaction of LPS and TLR4 complex. In the ELISA analysis, 25HC clearly inhibited the biotin‐LPS from binding to MD‐2 (Figure 4A). Fluorescence spectroscopy was further applied to observe the interaction of 25HC and MD‐2. As shown in Figure 4B, the emission spectra of rhMD‐2 had maximum emission wavelength at 450 nm. The fluorescence intensity of rhMD‐2 decreased gradually with the increasing concentrations of 25HC ranging from 1 μmol/L to 20 μmol/L, which indicated that 25HC has a high binding affinity with MD‐2. Next, we explored whether 25HC is sufficient to reduce cellular binding of LPS to MD‐2. Flow cytometry analysis demonstrated that 25HC decreased the binding of FITC‐LPS to the TLR4 complex in a dose‐dependent manner (Figure 4C). Accordingly, confocal microscopy analysis showed that the amounts of colocalization of LPS with MD‐2 were reduced by 25HC (Figure 4D). Finally, 25HC was shown be capable of docking into the hydrophobic pocket of MD‐2 on crystal structure (PDB ID: 2E59) via computer‐assisted simulations, overlapping with some binding sites of LPS antagonist lipid IVa. Moreover, 25HC‐binding prediction showed its proximity to Tyr‐102, Tyr‐65, Ile‐117 and Leu‐71 residues of MD‐2 in the most energetically favourable condition, forming a hydrogen bond with Tyr‐102 residue (Figure 4E). Collectively, these data indicate that 25HC binds with MD‐2 to prevent the interaction of LPS with the TLR4‐MD‐2 complex.

### 3.5 25HC attenuates LPS‐induced activation of Akt and NF‐κB pathway

**Figure 5:**
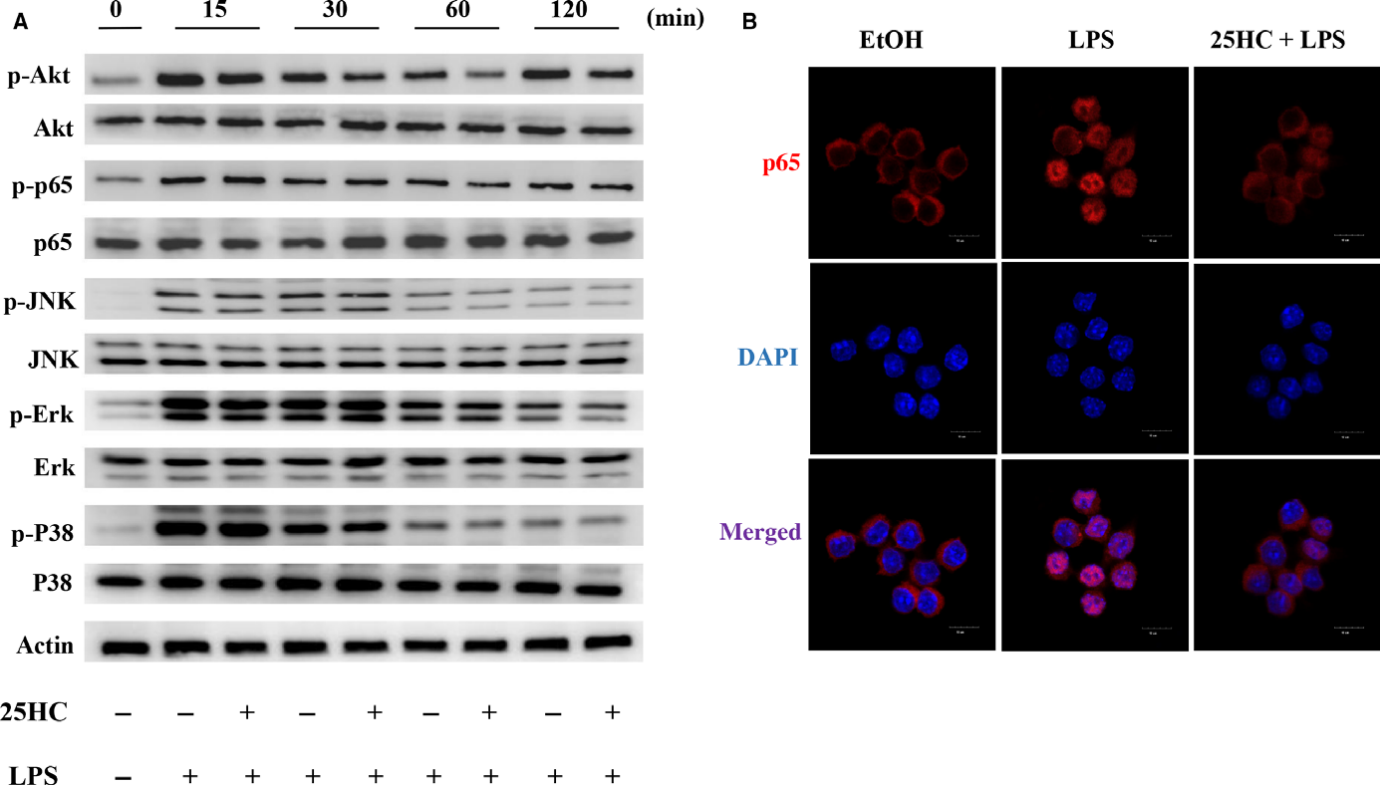
25HC attenuates LPS‐induced Akt and NF‐κb activation. RAW264.7 cells were preincubated with or without 25HC (10 μmol/L) for 2 h and then treated with 100 ng/mL LPS for the indicated time‐points. Cells treated with 0.1% ethanol (EtOH) were used as negative control. (A) Western blot analysis of phosphorylated and total proteins of Akt, p65, JNK, Erk and P38. (B) Nuclear translocation of p65 NF‐κB after 1 h LPS stimulation was detected by confocal fluorescence microscopy. NF‐κB p65 was shown red staining and nuclei in blue. Scale bar, 10 μm

**Figure 6:**
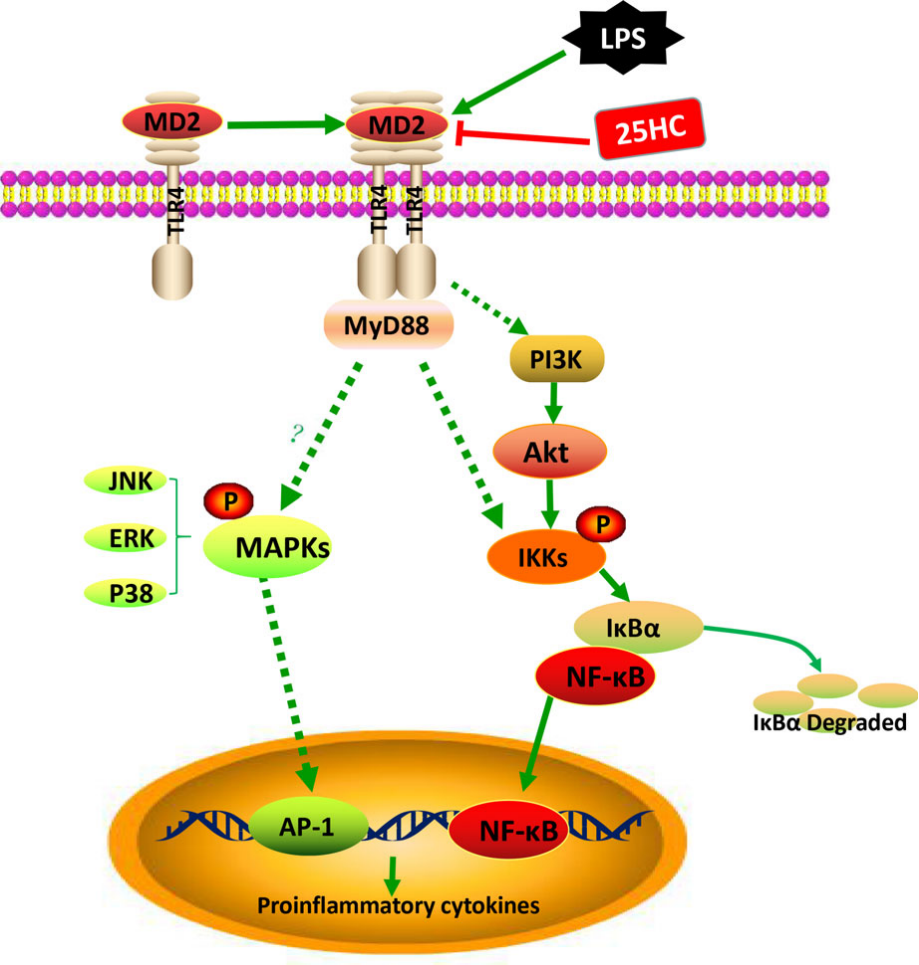
Proposed model of 25HC preventing LPS‐induced TLR4 signalling pathway. LPS binding to MD2 promotes the dimerization of TLR4/MD‐2. The conformational changes in the TLR4 induce the recruitment of intracellular adaptor proteins to activate the downstream signalling pathway. The MyD88‐dependent pathway involves activation of MAPK cascades and IKK (IκB kinase). Phosphorylation of IKKs leads to degradation of IκBα and the release of NF‐κB. Transcription factors such as NF‐κB, AP‐1 and etc. participate in driving gene expression of proinflammatory cytokines. The activated PI3K/Akt in the downstream of TLR4 induces nuclear translation of NF‐κB. 25HC could directly interact with MD2 to prevent LPS‐induced activation of Akt and NF‐κB signal pathway

To explore the effect of 25HC on the LPS‐initiated TLR4 signalling, we measured the downstream signal molecules of TLR4 in RAW264.7 cells. Pretreatment with 25HC partially decreased the LPS‐induced phosphorylation of Akt and NF‐κB, but it did not affect the up‐regulated phosphorylation of JNK, Erk and P38 MAPKs stimulated by LPS (Figure 5A). Confocal fluorescence microscopy revealed the nuclear translocation of NF‐κB p65 was suppressed by 25HC pretreatment as well (Figure 5B). These findings suggest that the ameliorative activity of 25HC on the LPS‐induced inflammatory response is at least partially mediated through the Akt/NF‐κB signalling in macrophages. The proposed model of 25HC preventing LPS‐induced TLR4 signalling pathway is shown as Figure 6.

## 4 DISCUSSION

ALI is a life‐threatening clinical condition with substantial mortality. To date, several clinical trials showed ineffectiveness of many pharmacological strategies in reducing ALI‐related mortality.^27^ Here, we demonstrate that as one of the oxysterols, 25HC has therapeutic effects on LPS‐induced ALI via reduction in the inflammatory responses. We further reveal that the underlying anti‐inflammatory mechanism of 25HC is mainly based on prevention of LPS binding to TLR4‐MD‐2 complex by its engagement with the hydrophobic pocket of MD‐2, which consequently impairs the activation of downstream Akt/NF‐κb signalling and dampens induction of inflammatory cytokines.

As major inflammatory cells in innate immunity, alveolar macrophages are activated by LPS via TLR4‐mediated signal transduction, leading to production of proinflammatory cytokines.^28^ Activated macrophages prompt the recruitment of circulating neutrophils into the site of lung by secreting abundant chemokines. The infiltration of neutrophils releases inflammatory mediators, including IL‐1β, IL‐6, IL‐8 and TNF‐α, which in turn enhances the responses of neutrophils and cause uncontrolled lung inflammation.^29^, ^30^ Therefore, modulation of excessive inflammation is an effective therapeutic strategy for ALI.

The oxysterol 25HC is synthesized by cholesterol 25‐hydroxylase (Ch25h), which is an interferon‐stimulated gene (ISG) strongly up‐regulated in macrophages after stimulation by TLR ligands.^19^, ^31^, ^32^ 25HC has been reported to repress the activation of sterol regulatory element‐binding proteins (SREBPs) to interfere with cholesterol biosynthesis.^33^ In addition, 25HC could activate orphan nuclear receptors such as liver X receptors (LXRs) and proliferation activator receptor gamma (PPARγ) to regulate lipid metabolism and inflammation.^34^, ^35^ However, the contribution of 25HC to inflammatory response still remains unclear. It has been reported that 25HC induced the release of proinflammatory cytokines such as IL‐8 and IL‐6 and amplify the response of TLR3 in airway epithelial cells via NF‐κB.^22^ 25HC was shown to enhance inflammatory signalling in bone marrow‐derived macrophages (BMDM) and expression of Ch25h exacerbated morbidity following influenza infection via AP‐1.^36^ Jang et al^37^ demonstrated that 25HC activated the NLRP3 inflammasome to increase IL‐1β level without affecting the IL‐6, leading to cerebral neuroinflammation. Rosklint et al^38^ observed that 25HC dramatically increased IL‐1β secretion with or without the addition of LPS in human macrophages, but it decreased IL‐6 production in LPS‐treated cells. However, Reboldi et al recently found that 25HC negatively regulated IL‐1β transcription through antagonizing SREBP processing in LPS‐activated BMDM. Ch25h knockout mice displayed low survival rate than wild‐type mice in septic shock.^23^ Here, we found 25HC at micromolar concentrations to effectively inhibit the expression of proinflammatory cytokines and chemokines in LPS‐treated RAW264.7 cells. The disparity from different works may be due to the different experimental conditions, such as the selected cell lines and periods of 25HC treatment.

The LPS‐triggered immune response requires its binding to MD‐2, the TLR4 coreceptor, on the cell surface.^39^, ^40^ MD‐2 is associated with the extracellular domain of TLR4 and directly recognizes the lipid chains of LPS. It contains a hydrophobic pocket for ligand binding that is sandwiched by two β antiparallel sheets. The internal surface of MD‐2 pocket is lined with hydrophobic residues, and the opening rims of the pocket were thick with positively charged residues that facilitate the LPS binding.^12^, ^41^ A study revealed MD‐2 to be a potential target for treating LPS‐induced toxic effects. MD‐2 knockout mice did not respond to LPS and thus showed comparatively few cytokines production induced by LPS.^42^ Lipid IVa and eritoran, a synthetic MD‐2 targeting lipid A analog, can antagonize LPS binding to the same site, leading to inhibition of TLR4 signalling.^10^, ^43^ In addition, some natural and synthetic chemicals which are structurally unrelated to bacterial LPS or lipid A have been found to prevent the engagement of LPS with MD‐2. The chalcone‐type flavonoids, including JSH (2′,4‐ dihydroxy‐6′‐isopentyloxychalcone) and xanthohumol (4,4′‐dihydroxy ‐5′‐isopentenyl‐2′ ‐methoxychalcone), were reported to inhibit TLR4‐mediated NF‐κB activation via directly antagonizing LPS binding to MD‐2.^41^, ^44^ Moreover, xanthohumol binding was located in the deep hydrophobic pocket of the MD‐2, adjacent to and hydrogen‐bonded with Tyr‐102. A chalcone derivative L2H21 also showed as a direct inhibitor of MD‐2 by binding to Tyr‐102 and Arg‐90 residues in the hydrophobic pocket of MD‐2.^24^ Here, we demonstrate that 25HC binds to MD‐2 with high affinity and inhibits the interaction between LPS and MD‐2. From the molecular docking results, 25HC is predicted to bind to the hydrophobic pocket of MD‐2 and in close proximity to the Tyr‐102, Tyr‐65, Ile‐117 and Leu‐71 residues, forming a hydrogen bond with Tyr‐102.

25HC was previously described as a potential ligand for LXR pathway, the activation of which inhibited proinflammatory genes expression in immune cells.^45^, ^46^ The other study showed 25HC decreased nuclear peroxisome proliferation activator receptor γ (PPARγ) and increased nuclear NF‐κB levels, which induced release of proinflammatory cytokines.^35^ NF‐κB is known as a major transcription factor participating in regulation of inflammatory cytokine generation.^47^ Phosphoinositide 3‐kinase downstream kinase Akt has been shown to be involved in modulating NF‐κB activation.^48^ Additionally, the MAPK pathway also plays a significant role in TLR4‐mediated inflammation.^49^ Our current data indicate that 25HC interferes with LPS binding to TLR4 complex and inhibits the activation of Akt/NF‐κB signal pathway, but does not affect the MAPK pathway. Considering the multiple effects of 25HC, the downstream signalling of TLR4 is likely selectively inhibited by 25HC.

In conclusion, 25HC modulates excessive inflammatory responses and protects against LPS‐induced ALI via directly targeting MD‐2. This study offers a therapeutic strategy against ALI through inhibition of MD‐2‐mediated inflammatory signalling.

## CONFLICT OF INTEREST

The authors declare that they have no conflicts of interest.
